# Change in left inferior frontal connectivity with less unexpected harmonic cadence by musical expertise

**DOI:** 10.1371/journal.pone.0223283

**Published:** 2019-11-12

**Authors:** Chan Hee Kim, June Sic Kim, Yunhee Choi, Jeong-Sug Kyong, Youn Kim, Suk Won Yi, Chun Kee Chung

**Affiliations:** 1 Interdisciplinary Program in Neuroscience, Seoul National University College of Natural Science, Seoul, Korea; 2 Department of Brain and Cognitive Science, Seoul National University College of Natural Science, Seoul, Korea; 3 Research Institute of Basic Sciences, Seoul National University, Seoul, Korea; 4 Medical Research Collaborating Center, Seoul National University College of Medicine, Seoul National University Hospital, Seoul, Korea; 5 Neuroscience Research Institute, Seoul National University Medical Research Center, Seoul, Korea; 6 Audiology Institute, Hallym University of Graduate Studies, Seoul, Korea; 7 Department of Music, School of Humanities, The University of Hong Kong, Hong Kong, China; 8 College of Music, Seoul National University, Seoul, Korea; 9 Western Music Research Institute, Seoul National University, Seoul, Korea; 10 Department of Neurosurgery, Seoul National University Hospital, Seoul, Korea; Anadolu University, TURKEY

## Abstract

In terms of harmonic expectancy, compared to an expected dominant-to-tonic and an unexpected dominant-to-supertonic, a dominant-to-submediant is a less unexpected cadence, the perception of which may depend on the subject’s musical expertise. The present study investigated how aforementioned 3 different cadences are processed in the networks of bilateral inferior frontal gyri (IFGs) and superior temporal gyri (STGs) with magnetoencephalography. We compared the correct rate and brain connectivity in 9 music-majors (mean age, 23.5 ± 3.4 years; musical training period, 18.7 ± 4.0 years) and 10 non-music-majors (mean age, 25.2 ± 2.6 years; musical training period, 4.2 ± 1.5 years). For the brain connectivity, we computed the summation of partial directed coherence (PDC) values for inflows/outflows to/from each area (sPDC_i_/sPDC_o_) in bilateral IFGs and STGs. In the behavioral responses, music-majors were better than non-music-majors for all 3 cadences (*p* < 0.05). However, sPDC_i_/sPDC_o_ was prominent only for the dominant-to-submediant in the left IFG. The sPDC_i_ was more strongly enhanced in music-majors than in non-music-majors (*p* = 0.002, Bonferroni corrected), while the sPDC_o_ was vice versa (*p* = 0.005, Bonferroni corrected). Our data show that music-majors, with higher musical expertise, are better in identifying a less unexpected cadence than non-music-majors, with connectivity changes centered on the left IFG.

## Introduction

Humans exposed to Western tonal music can expect a tonic after a dominant at the end of musical pieces. Compared to a dominant-to-tonic of an “expected” condition, a dominant-to-supertonic is an “unexpected” condition, therefore eliciting an early right anterior negativity (ERAN) in previous studies [1–3]. The ERAN for the unexpected condition is commonly observed both in musicians and non-musicians alike [3, 4]. In contrast, the dominant-to-submediant lies in between the “expected” and “unexpected”, thus could be called as “less unexpected”, without eliciting the ERAN of the unexpected condition [2, 3]. The conditional probability [5], of the different chords following the dominant is 0.752 for tonic, 0.106 for submediant, and 0.043 for supertonic.

The “Do Mi” in a tonic comprising of “Do Mi Sol” is also included in a submediant of “Ra Do Mi”. Since a dominant-to-submediant may sound similar to a dominant-to-tonic, it is called a deceptive cadence [6, 7]. In a musical piece, the deceptive cadence functions as delaying the eventual resolution to a tonic in an authentic cadence, adding suspense, and leading to longer sustained anticipation for the moment of resolution. However, the identification of dominant-to-submediant may depend on people’s musical expertise, since the difference between dominant-to-tonic and dominant-to-submediant is subtle.

**Fig 1:**
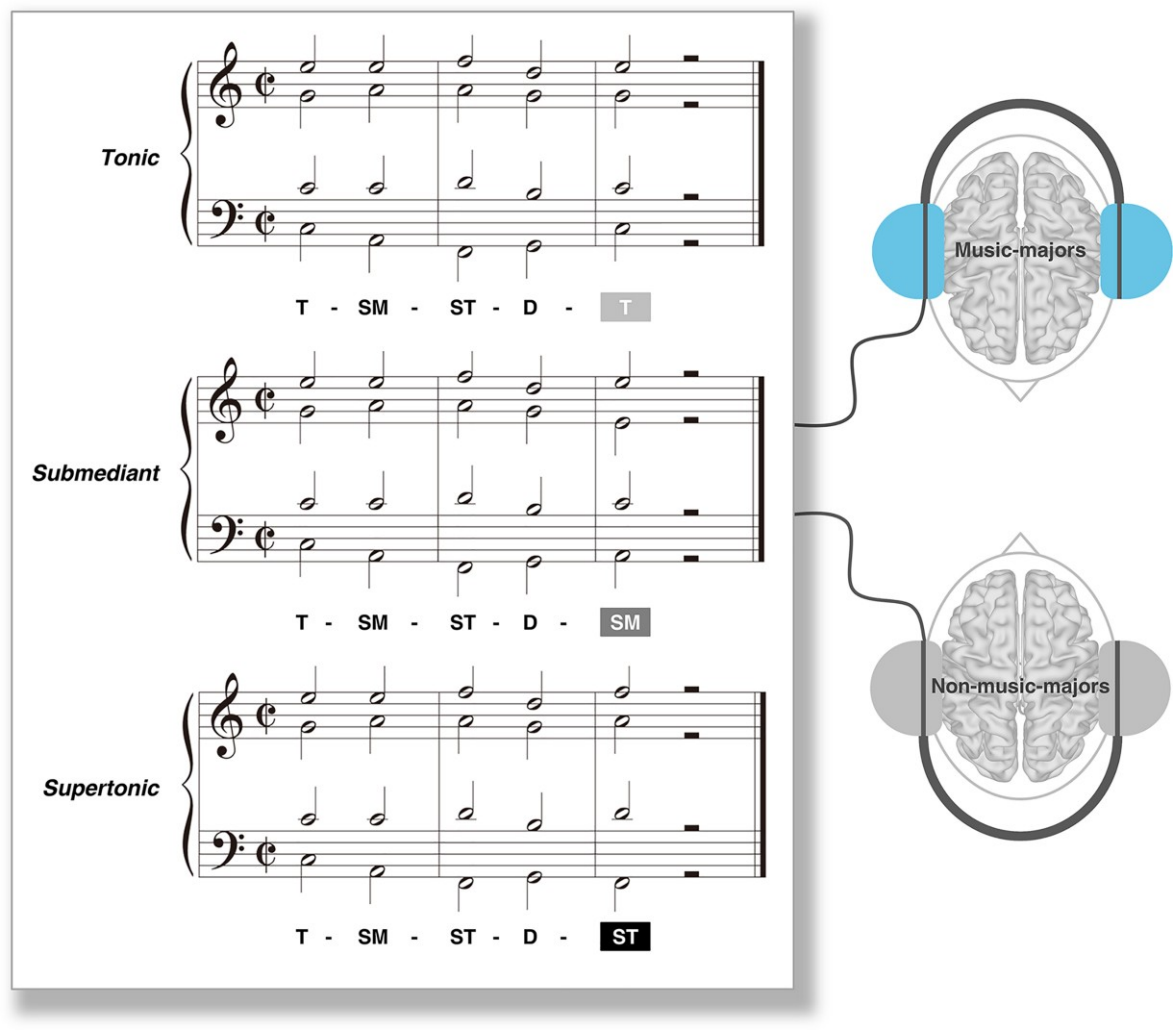
Musical stimuli. Three conditions were presented randomly to music-majors and non-music-majors in all experiments. The three conditions had the different final chords of tonic (T), submediant (SM), and supertonic (ST) following an identical sequence of T—SM—ST—dominant (D), respectively. When the sequence of 4^th^ and 5^th^ chords is dominant-to-tonic (D—T, top), it is an authentic cadence which is classified as a conclusive cadence. The dominant-to-submediant (D—SM, middle) is a deceptive cadence which is classified as a progressive cadence (middle). The dominant-to-supertonic (D—ST, bottom) is not a cadential form. After D, the T is most expected, the ST is most unexpected, and the SM is less unexpected. The three conditions were named *Tonic*, *Submediant*, and *Supertonic*, respectively, according to the final chord.

The hypothesis examined in the present study is whether people with musical expertise (music-majors) are better in identification of dominant-to-submediant with specific brain connectivity change than people without it (non-music-majors). We measured behavioral response and brain connectivity with 3 different harmonic cadences of dominant-to-tonic, dominant-to-submediant, and dominant-to-supertonic in 4^th^ and 5^th^ chords in sequences of five chords (Fig 1).

For the brain connectivity, we used partial directed coherence (PDC) [8], multivariate measurement of effective connectivity, estimating causal relationship between multivariate time series of 4 regions of interest (ROIs) comprised of bilateral inferior frontal gyri (IFGs) and the superior temporal gyri (STGs). We compared the summation of PDC values (sPDC) for the inflow and outflow signals in the 4 ROIs (bilateral IFGs and STGs), for 3 conditions (*Tonic*, *Submediant*, and *Supertonic*) for 2 groups (music-majors and non-music-majors).

## Materials and methods

### Ethics statements

The present study was approved by the Institutional Review Board of the Clinical Research Institute, Seoul National University Hospital (H-1001-020-306). Prior to the experiments, the participants provided informed consent in written form. All experiments were conducted in accordance with the ethical guidelines. In the present study, we used the data of 5 music-majors and 5 non-music-majors in the data set of our previous studies that was already published [2, 3]. The other participants were newly recruited. The data sets of our previous and present studies are partially overlapped, and the experimental procedure is also the same of them, however, the present study is an independent study applying novel hypotheses and analyses.

### Participants

The 19 participants were 9 music-majors (mean age, 23.5 ± 3.4 years) and 10 non-music-majors (mean age, 25.2 ± 2.6 years). The music-majors majored in musical instruments at music colleges, and each trained for at least fifteen years (musical training period, 18.7 ± 4.0 years). Non-music-majors were not music majors (musical training period, 4.2 ± 1.5 years). All participants were females and right handed, and had normal hearing.

### Experimental procedure

The whole experiment was performed in a magnetically shielded MEG room, which minimized background noise out of musical stimuli. All music-majors and non-music-majors participated in 6 MEG recording sessions and 3 behavioral sessions. The three conditions of *Tonic*, *Submediant*, and *Supertonic* were presented randomly in all experiments. In each session, the participants listened to the five chord sequences of each condition. The individual sequence was 3,600 ms long. They were made up of five chords, 3,000 ms, and a break, 600 ms. In the MEG sessions, the participants were asked to detect a staccato chord (37.5 ms, 1/16 of other chords) randomly presented in the third to fifth chord in each sequence, and responded using a computer mouse. Each session included 100 sequences (10 staccato sequences). In the behavioral sessions, the participants were asked to categorize the 108 sequences (36 sequences per condition) in each session by type of condition, using a keypad. The whole experiment took about two hours, including preparation.

The three conditions were transposed to twelve major keys, and were randomly shuffled in each session. The musical stimuli were constructed with the piano timbre (Bösendorfer 290 Imperial grand) in Grand 3 (Steinberg Media Technologies, Hamburg, Germany) software. The wave files (sampling rate: 44.1 KHz; 16-bit; stereo; windows PCM) normalized the intensity by Cool Edit Pro 2.1 (Syntrillium Software Corporation, Phoenix, AZ, USA), and were recorded at 100 BPM using Cubase 5 (Steinberg Media Technologies, Hamburg, Germany) software. The sound pressure level in the musical stimuli was 65 dB, which was presented into MEG-compatible tubal insert earphones (Tip-300, Nicolet, Madison, WI, USA) by the STIM^2^ (Neuroscan, Charlotte, NC, USA).

### MEG data acquisition and preprocessing

Using a 306-channel whole-head MEG System (Elekta NeuroMag VectorView^™^, Helsinki, Finland), MEG signals (600.615 Hz sampling rate, 0.1–200 Hz filter) were recorded in a magnetically shielded room. Electrooculograms (EOG) and electrocardiograms were also simultaneously recorded. In preprocessing, the environmental and movement noise of raw MEG signals was removed by the temporal Signal-Space Separation algorithm in MaxFilter 2.1.13 (Elekta Neuromag Oy, Helsinki, Finland) [9, 10]. Periods/Epochs containing EOG artifacts were manually rejected based upon visually inspection. The MEG data was filtered with a 1-20Hz band-pass filter.

### Source estimation

**Fig 2:**
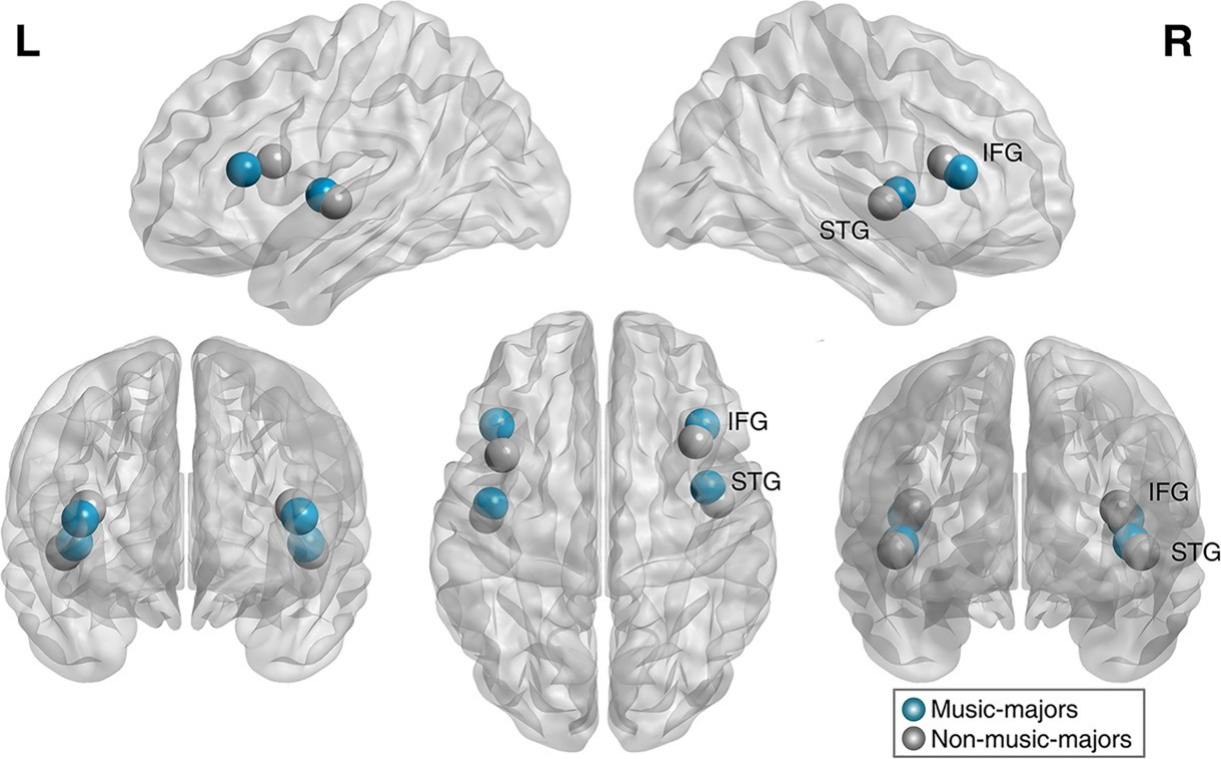
The grand mean dipole source for music-majors and non-music-majors. The dipole source locations for the bilateral IFGs and STGs were not significantly different between the music-majors and the non-music-majors (Mann–Whitney U test, *p* > 0.05 in all cases, uncorrected for multiple comparisons). The Talairach coordinates of mean dipole sources for the 4 ROIs in two groups were visualized using BrainNet Viewer (http://nitrc.org/projects/bnv/). See the Materials and Methods section for Talairach coordinates of music-majors and non-music-majors.

The signals for multiple equivalent current dipoles in the bilateral IFGs and STGs were extracted from epochs of 400 ms after the onset of the final chord (tonic, submediant, and supertonic) in each condition using BESA 5.1.8.10 (MEGIS Software GmbH, Gräfelfing, Germany). For the dipole locations of the bilateral IFGs and STGs (4 ROIs), there was no significant difference between the music-majors and the non-music-majors (Mann–Whitney U test, *p* > 0.05 in all cases, uncorrected for multiple comparisons; see Fig 2). The Talairach coordinates (x, y, and *z*; millimeters) in the left STG were -44.3, -7.1, and 3.2 for music-majors, and were -45.9, -11.6, and 0.6 for non-music-majors (Euclidian distance: 5.4 mm). The right STG was 41.3, -0.5, and 3.6 for music-majors, and were 45, -5.6, and 0.1 for non-music-majors (Euclidian distance: 7.2 mm). The left IFG was -41.9, 24.1, and 13.3 for music-majors, and were -39.8, 12.4, and 16.6 for non-music-majors (Euclidian distance: 12.3 mm). The left IFG was 39.1, 23.9, and 12.3 for music-majors, and were 36.4, 17.8, and 16.1 for non-music-majors (Euclidian distance: 7.7 mm).

### PDC analysis

The effective connectivity between the bilateral IFGs and STGs was computed by PDC [8]. The PDC is a measure used to identify causality between two signals in the frequency domain, and for the concept of Granger causality in a time domain, which is derived from the multivariate autoregressive (MVAR) model as

$$X(n)=\sum_{k=1}^{p}A_{k}X(n-k)+E(n)$$ (1)

where X(n) is the data vector X of M time series, X(n) = [*X*_1_(*n*),*X*_2_(*n*),*X*_3_(*n*),…,*X*_*M*_(*n*)]^*N*^ (M = 4; the number of ROIs in the present study), and E(n) is multivariate uncorrected noise. *A*_*k*_ is N×N coefficient matrix. *P* is the model order, which was determined by Akaike’s information criterion.

We examined the causal relationship between the four ROIs. To evaluate the property in the frequency band, Eq(1) is transposed into the frequency domain as

$$A(f)=I-\sum_{k=1}^{p}A_{k}\;e^{-2\pi ikf}$$ (2)

where A(*f*) is the Fourier transform of coefficient matrix *A*_*k*_, and *I* is the identity matrix. The model order (*p*) was determined by Bayesian information criteria. Thus, the PDC value, *γ*_*ji*_, from ROI *j* to ROI *i* at frequency *f* is defined as

$$\gamma_{ji}(f)=\frac{{\bar{A}}_{ij}(f)}{\sqrt{{\bar{a}}_{j}^{H}∙{\bar{a}}_{j}(f)}}$$ (3)

where ${\bar{A}}_{ij}(f)$ is the *i*th and *j*th elements of matrix *A*_*k*_, and H is the Hermetian operator. In addition, the inflow to ROI *γ*_*ji*_(*f*) is emphasized because the PDC value is normalized with respect to the outflow from ROI *j*. The PDC value is “1” when signal to ROI *i* comes from the outflow signal from ROI *j*. If there is no signal flow from ROI *j* to ROI *i*, the value is close to “0”. The PDC values for twelve connections (_4_P_2_; 12 permutations of four ROIs) and three conditions in individual participants were averaged over 1-20Hz frequency range. The mean PDC values were normalized by Fisher’s Z-transformation. The value of Z-transposed PDC was over the range of 0–1.

Based on the twelve Z-transposed mean PDC values for four ROIs of the bilateral IFGs and STGs, we computed the summation of the Z-transposed mean PDC values for the inflows and outflows in individual ROIs (left IFG, right IFG, left STG, and right STG); i.e. the inflow in the left IFG was the summation of the Z-transposed mean PDC values of “Left STG → Left IFG”, “Right STG → Left IFG”, and “Right IFG → Left IFG” among twelve connections, while the outflow in the left IFG was the summation of “Left IFG → Left STG”, “Left IFG → Right STG”, and “Left IFG → Right IFG”. Hereafter, we used the “sPDC_i_” as the term referring to the summation of the Z-transposed mean PDC values for the inflows and the “sPDC_o_” as the term referring to the summation of the Z-transposed mean PDC values for the outflows.

### Statistics

The data of the dipole location and behavioral responses did not follow a normal distribution. We conducted nonparametric analysis of the Mann–Whitney U test and Friedman test to test the difference between groups and conditions for the dipole location and behavioral responses. The sPDC_i_/sPDC_o_ was tested by four-way repeated measures ANOVA with the factors of Condition (*Tonic*, *Submediant*, and *Supertonic*) × Group (music-majors and non-music-majors) × Site (IFG and STG) × Hemisphere (left hemisphere and right hemisphere) and three-way repeated measures ANOVA with the factors of Condition × Group × Flow (inflow and outflow). In all ANOVAs for the sPDC_i_ and sPDC_o_, the Greenhouse-Geisser’s correction was applied when the Mauchly Sphericity test was significant (*p* < 0.05). The *P*-values (*p* < 0.05) for multiple comparisons in the results of all statistical analyses were adjusted based on the Bonferroni test. The statistical analyses were performed by SPSS 21.0 software (IBM, Armonk, NY, USA).

## Results

### Behavioral tests

**Fig 3:**
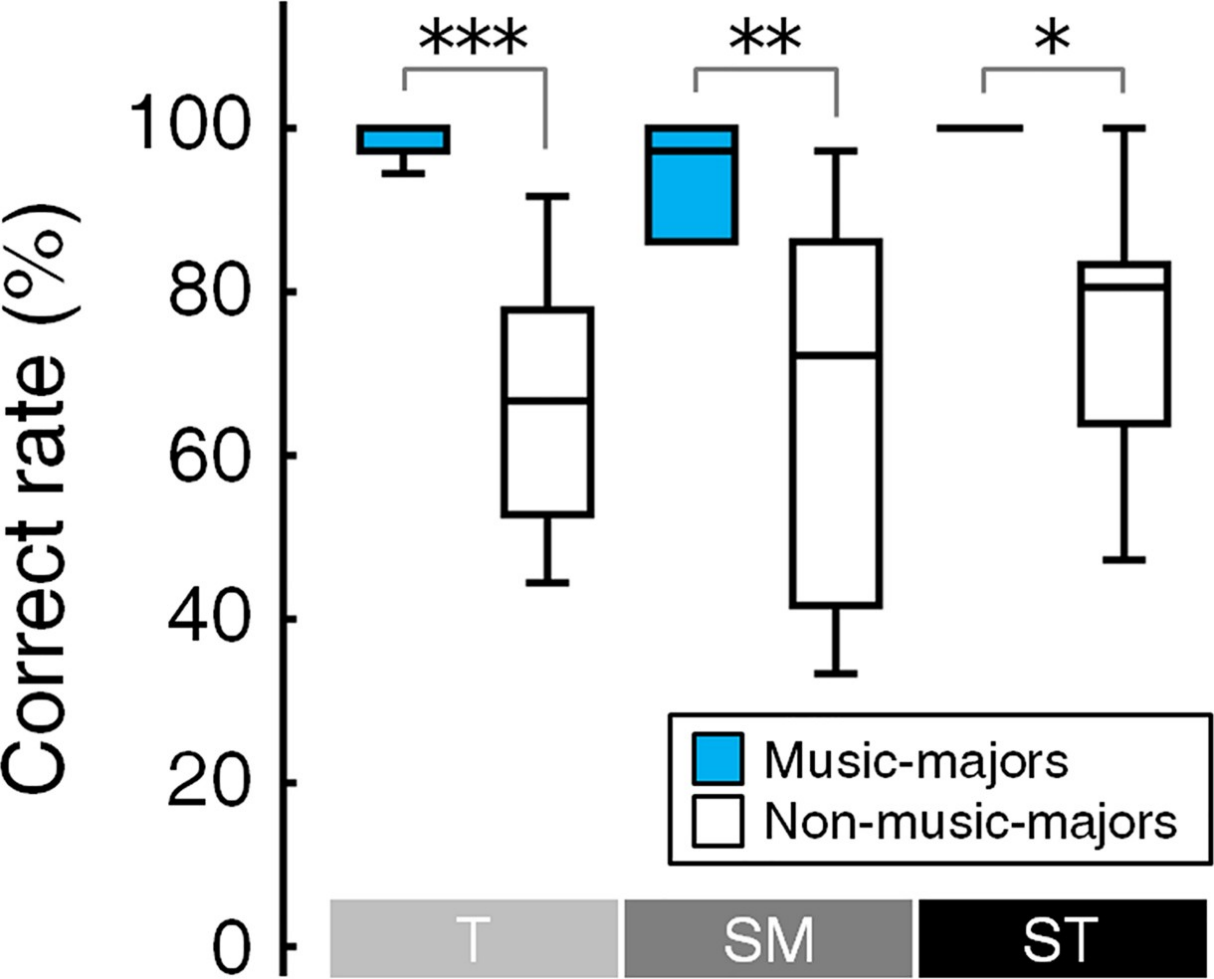
Group difference in the correct rate. Music-majors responded more correctly than non-music-majors for all conditions. Correct rates for the *Tonic*, *Submediant*, and *Supertonic* were higher in music-majors than in non-music-majors (Mann–Whitney U test, * *p* < 0.05, ** *p* < 0.01, *** *p* < 0.001, Bonferroni corrected). See also the Results section for the details of statistical results. In each box plots, the box represents 1^st^ and 3^rd^ quartiles, the line is the median value, and the whisker represents the most extreme non-outlier value. T = *Tonic*, SM = *Submediant*, and ST = *Supertonic*.

In the behavioral experiment, the music-majors responded more correctly and more quickly than the non-music-majors (Fig 3). The correct rate was different between the two groups under all conditions (Mann–Whitney U test, *Tonic*, Z = -3.700, *p* = 0.0006; *Submediant*, Z = -2.8, *p* = 0.012; *Supertonic*, Z = -3.016, *p* = 0.006, Bonferroni corrected). The music-majors’ mean correct rates were over 90% in all conditions, whereas the non-music-majors’ mean correct rates were under 80%.

Additionally, for the behavioral results on detecting the staccato sequence in the MEG experiment, no participant incorrectly responded more than 5%, which was the same for music-majors and non-music-majors (*p* > 0.5 in all cases). All participants concentrated on the musical stimuli during the MEG experiment.

### PDC

Four-way repeated measures ANOVAs with the factors of Condition × Group × Site × Hemisphere were tested for both inflow (sPDC_i_) and outflow (sPDC_o_). For both inflow (sPDC_i_) and outflow (sPDC_o_), the interactions of Condition × Group × Site × Hemisphere were significant [Inflow, *F*(2, 136) = 5.963, *p* = 0.003; Outflow, *F*(2, 136) = 5.681, *p* = 0.004]. See the S1 Table the details of statistical results of four-way repeated measures ANOVAs.

**Fig 4:**
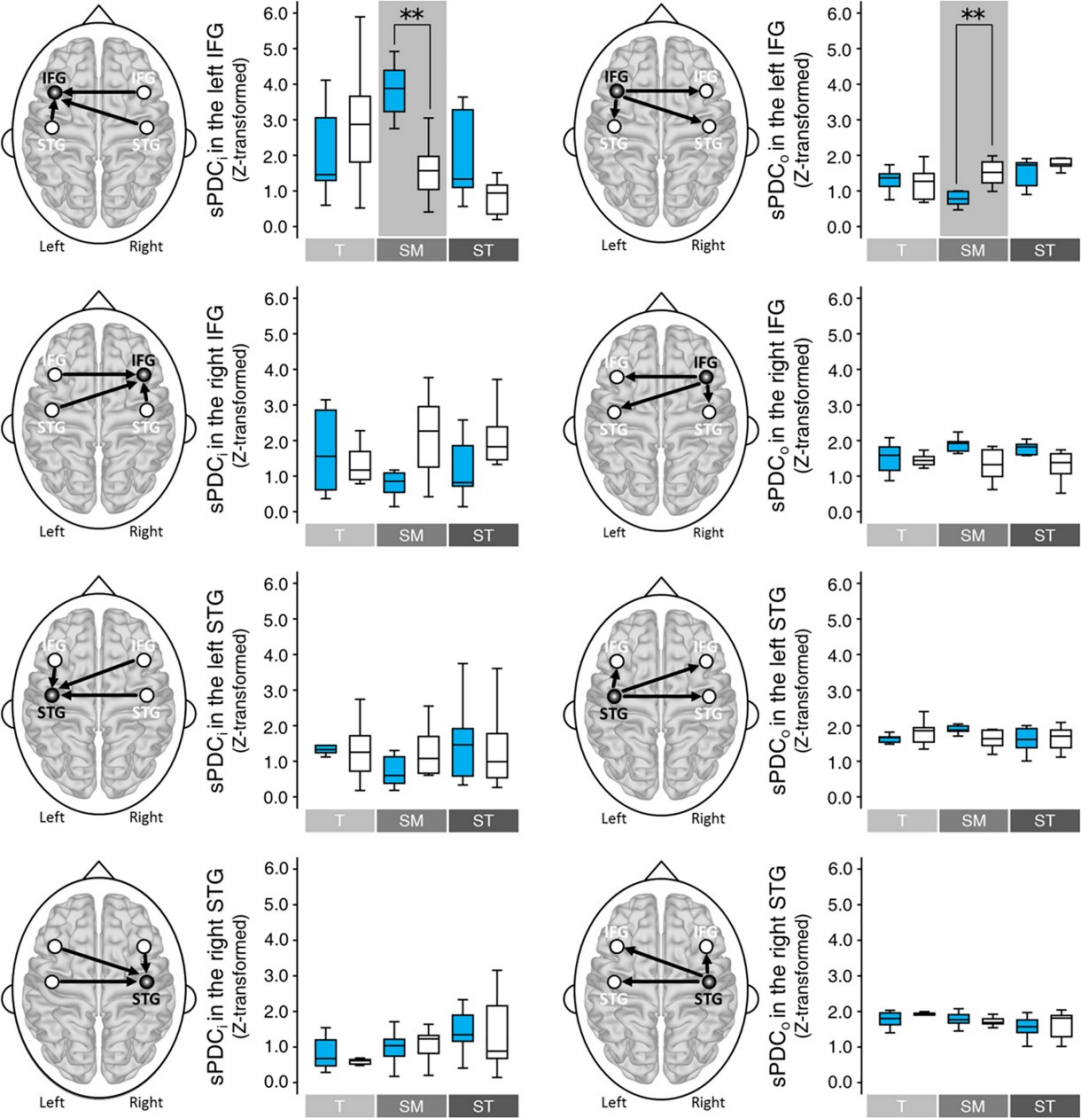
Group difference in the PDC. Group difference was observed only for the *Submediant* in both inflow and outflow of the left IFG (gray shaded boxes). The sPDC_i_ for the *Submediant* was higher in music-majors than in non-music-majors, while the sPDC_o_ for the *Submediant* was lower in music-majors than in non-music-majors (** *p* < 0.01, Bonferroni corrected). See also the S2 and S6 Tables for the detail of statistical results. In each box plots, the box represents 1^st^ and 3^rd^ quartiles, the line is the median value, and the whisker represents the most extreme non-outlier value. T = *Tonic*, SM = *Submediant*, and ST = *Supertonic*.

In *post hoc*, the Group difference was only observed in the left IFG, in both inflow and outflow, which was observed only in the *Submediant* among the three conditions (Fig 4). The sPDC_i_ for music-majors was much higher than it was for non-music-majors [*t*(17) = 4.934, *p* = 0.002, Bonferroni corrected], whereas the sPDC_o_ for music-majors were much lower than it was for non-music-majors [*t*(17) = -4.391, *p* = 0.005, Bonferroni corrected] (See also the S2 and S6 Tables for the details of statistical results).

**Fig 5:**
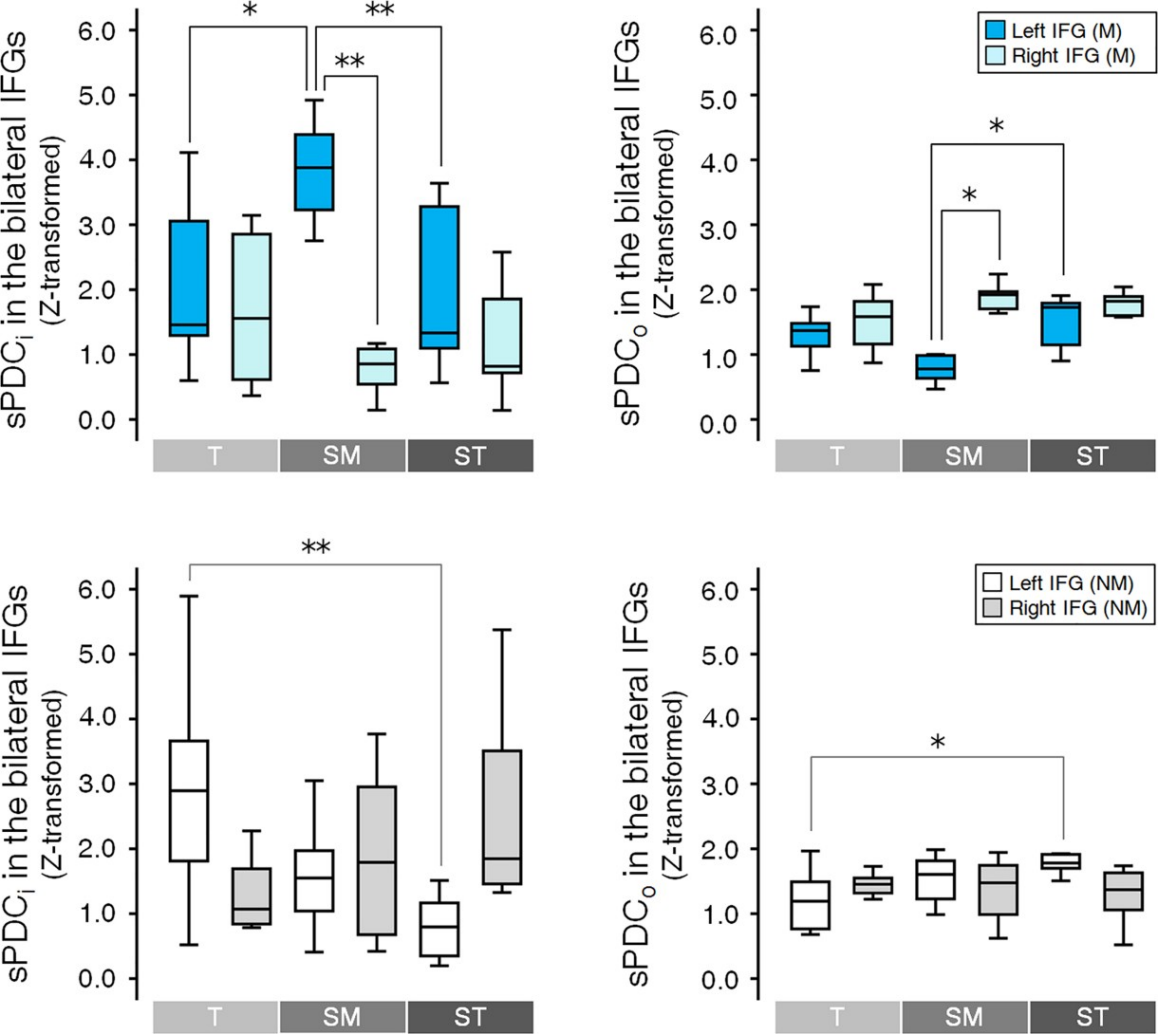
Hemisphere and condition differences in the PDC. The hemispheric difference was observed only for the *Submediant* of music-majors in the IFG (See the S3 and S6 Tables for the detail of statistical results). The sPDC_i_ was higher for the left IFG than for the right IFG, and the sPDC_o_ was lower for the left IFG than for the right IFG (* *p* < 0.05 and ** *p* < 0.01, Bonferroni corrected). Condition difference was observed in both groups (See also the S4 and S6 Tables for the detail of statistical results). In music-majors, the sPDC_i_ was significantly higher for the *Submediant* than for the other conditions. In the sPDC_o,_ the *Submediant* was significantly lower than the *Supertonic*. In non-music-majors, the difference between the conditions was observed between the *Tonic* and the *Supertonic*. The sPDC_i_/sPDC_o_ was higher/lower for the *Tonic* than for the *Supertonic*. The box represents 1^st^ and 3^rd^ quartiles, the line is the median value, and the whisker represents the most extreme non-outlier value. T = *Tonic*, SM = *Submediant*, ST = *Supertonic*, M = music-majors, NM = Non-music-majors.

Also *post hoc* for the Hemisphere difference was only observed in the *Submediant* in both inflow and outflow (Fig 5). Only in the bilateral IFG of the music-majors, the sPDC_i_ was higher in the left IFG than in the right IFG [*t*(8) = 5.262, *p* = 0.009, Bonferroni corrected], and the sPDC_o_ was lower in the left IFG than in the right IFG [*t*(8) = -4.889, *p* = 0.015, Bonferroni corrected] (See also the S3 and S6 Tables for the details of statistical results). Except for the two, there were no significant differences between hemispheres.

In the Fig 5, the difference between conditions was tested by post hoc one-way repeated measures ANOVA. The effect of Condition was only significant for the left IFG among 4 ROIs [Inflow, Music-majors, *F*(2, 16) = 7.583, *p* = 0.039, Bonferroni corrected; Outflow, Music-majors, *F*(2, 16) = 8.969, *p* = 0.020, Bonferroni corrected; Inflow, Non-music-majors, *F*(2, 18) = 9.825, *p* = 0.010, Bonferroni corrected; Outflow, Non-music-majors, *F*(2, 18) = 7.987, *p* = 0.026, Bonferroni corrected] (See also the S4 and S6 Tables for the details of statistical results). In music-majors, the sPDC_i_ for the *Submediant* was higher than for the other conditions [*Tonic*, *t*(8) = -3.153, *p* = 0.041, Bonferroni corrected; *Supertonic*, *t*(8) = 4.509, *p* = 0.006, Bonferroni corrected], and the sPDC_o_ was lower for the *Submediant* than for the *Supertonic* [*t*(8) = -4.143, *p* = 0.010, Bonferroni corrected]. In non-music-majors, the sPDC_i_ for the *Tonic* was higher than for *Supertonic* [*t*(9) = 4.151, *p* = 0.007, Bonferroni corrected], and the sPDC_o_ for the *Tonic* was lower than for the *Supertonic* [*t*(8) = -3.887, *p* = 0.011, Bonferroni corrected]. There were no significant differences in the other pairs.

**Fig 6:**
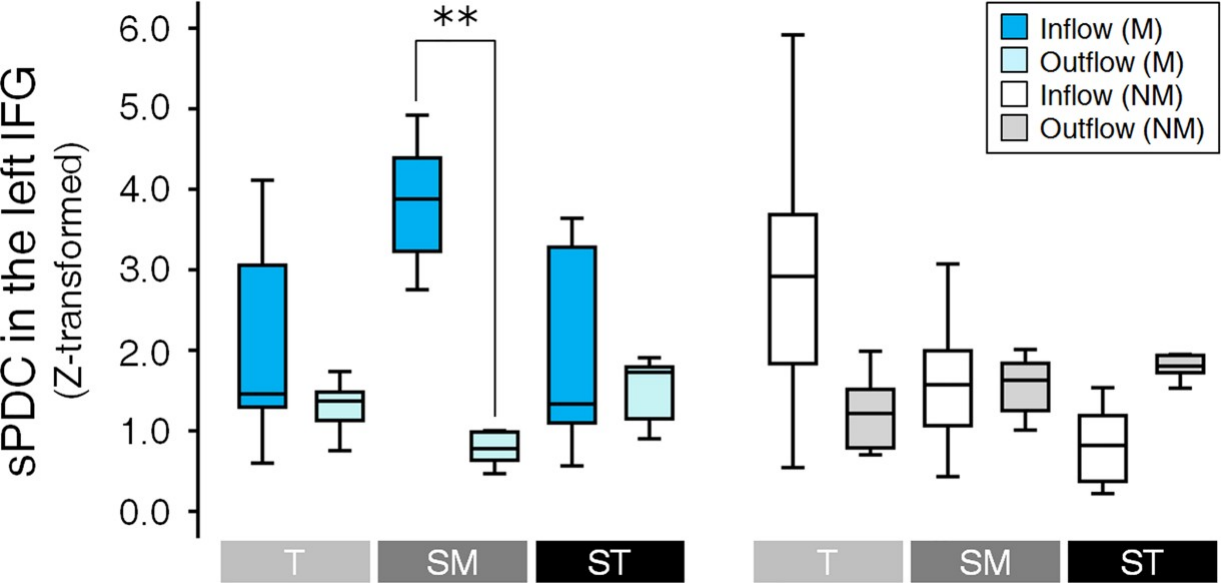
Flow difference in the PDC. In three-way repeated measures ANOVAs with the factors of Condition × Group × Flow, the interaction of Condition × Group × Flow was only significant for the left IFG [*F*(2, 68) = 14.661, *p* = 0.00002, Bonferroni corrected]. In the left IFG, the difference between sPDC_i_ and sPDC_o_ was only observed in the *Submediant* for music-majors (** *p* < 0.01, Bonferroni corrected). The sPDC_i_ was higher than the sPDC. See also S5 and S6 Tables for the detail of statistical results. The box represents 1^st^ and 3^rd^ quartiles, the line is the median value, and the whisker represents the most extreme non-outlier value. T = *Tonic*, SM = *Submediant*, ST = *Supertonic*, M = music-majors, and NM = Non-music-majors.

Flow difference was tested by separate models of three-way repeated measures ANOVAs with the factors of Condition × Group × Flow. Among the 4 ROIs, the interaction of Condition × Group × Flow was only significant for the left IFG [*F*(2, 68) = 14.661, *p* = 0.00002, Bonferroni corrected] (See also S5 and S6 Tables for the details of statistical results). In *post hoc* for the left IFG, the difference between sPDC_i_ and sPDC_o_ was only significant for the *Submediant* of music-majors [*t*(8) = 5.929, *p* = 0.002, Bonferroni corrected]. In the left IFG, the sPDC_i_ of the *Submediant* was higher than the sPDC_o_ (Fig 6). In non-music majors, the significant Flow differences were not observed.

Among 4 ROIs, for the right IFG, the interaction of Group × Flow was also significant [*F*(1, 34) = 11.583, *p* = 0.008, Bonferroni corrected] (See also S5 and S6 Tables for the details of statistical results). Music-majors showed the difference between sPDC_i_ and sPDC_o_ in the *Submediant*, unlike non-music-majors. The sPDC_i_ for the *Submediant* was significantly lower than its sPDC_o_ [*t*(8) = -3.784, *p* = 0.032, Bonferroni corrected]. In non-music-majors and the other condition pairs of music-majors, there were no significant differences. For the left and right STG, the Flow effects were only significant [Left STG, *F*(1, 34) = 12.196, *p* = 0.005, Bonferroni corrected; Right STG, *F*(1, 34) = 14.477, *p* = 0.002, Bonferroni corrected].

Accordingly, the group difference between music-majors and non-music-majors was only observed in the sPDC_i_ and sPDC_o_ for the *Submediant* in the left IFG. Moreover, the sPDC_i_ and sPDC_o_ for the *Submediant* in the left IFG were extremely prominent for the Condition, Hemisphere, and Flow factors. Even though the sPDC_i_ and sPDC_o_ for the *Tonic* of non-music-majors in the left IFG showed the significant differences between the conditions, it was distinct from the result of music-majors for the *Submediant*.

## Discussion

Music-majors were better than non-music-majors for all conditions in the behavioral experiment. However, consistent with our hypothesis, the difference between groups was only observed in the sPDC_i_/sPDC_o_ for the *Submediant* and the left IFG. Moreover, for music-majors, the inflows to the left IFG were strongly enhanced compared to the outflows from the left IFG. This indicates that the information for the *Submediant* collected from other areas (right IFG and bilateral STGs) is centered on the left IFG, where the information would be analyzed and integrated. These results imply that the sPDC_i_/sPDC_o_ in the left IFG was specialized in the processing of a deceptive cadence in the *Submediant*, and it depended on musical expertise.

The IFG and STG are well known to be associated with the processing of, and syntax of harmony and melody [2, 3, 11–18]. Although syntax in music is dominantly processed in the right IFG [11], the left IFG is prerequisite in the processing of musical syntax [19],. The left IFG is also highly activated by complexities in syntax [20]. However, it has not been studied how the deceptive cadence is processed in the network of bilateral IFGs and STGs. The present study demonstrated that the IFG plays a pivotal role in connections of bilateral IFGs and STGs, which was only related with the processing of the *Submediant* among three conditions.

Only in music-majors, the inflow (sPDC_i_) for the *Submediant* was higher in the left IFG than in right IFG, which was inversed in the outflow (sPDC_o_). Also, the inflow in the left IFG for music-majors was much higher than its outflow, whereas, the inflow was lower than the outflow in the right IFG. These results imply that when processing the *Submediant* music-majors have used the left hemisphere of the IFG much more than non-music-majors. The quantity of information sending to the left IFG is also relatively more than the right IFG.

Previous studies have reported that the left hemisphere of music-majors is something different by musical knowledge and training. The left IFG is associated with the different strategies of musicians such as pitch labeling [14]. In the melody processing, the activation of the left IFG related with the working memory is increased in musicians [21]. In terms of the whole brain, the musician’s brain reveals left hemisphere asymmetry during perceiving harmony [22]. Furthermore, musical training affects connectivity between the regions as well as activation in each region. The connectivity in peri-sylvian areas related with pitch processing is more increased in musicians with absolute pitch [23, 24]. The listening strategies of musicians are different from those of non-musicians, which are revealed as a left hemispheric dominance in functional connectivity between the brain regions [25]. Further extending previous studies, the present study revealed that the music-majors with musical knowledge of the deceptive cadence of dominant-to-submediant had left hemispheric asymmetry in connectivity when processing the *Submediant*.

The *Submediant* of dominant-to-submediant is an unexpected cadence compared with the *Tonic* of dominant-to-tonic in the levels of harmonic expectancy [26, 27]. Also it is a deceptive cadence [28]. The conditional probability [5], based on the harmonic expectancy, of the *Submediant* was less expected than the most expected *Tonic* (only about 1/7); when the sum of the conditional probability for six cases according to the different chords following a dominant is 1, tonic is 0.752, submediant is 0.106, subdominant is 0.053, mediant is 0.045, supertonic is 0.043, and subtonic is 0.001. However, the *Submediant* was not the unexpected condition in our present study, unlike the unexpected *Supertonic* with the conditional probability of 1/17 for the *Tonic* [2, 3]. Moreover, the group difference in the sPDC for the *Submediant* did not reflect the sensitivity of musical training for the processing of harmonic expectancy [29, 30]. If the sPDC for the *Submediant* was elicited by unexpected cadence, the sPDC and group difference would be more strongly prominent for the *Supertonic*. Thus, for *Submediant*, the relative increase in sPDC_i_ and the decrease in sPDC_o_ in the left IFG would not reflect harmonic expectancy in the dominant-to-submediant.

A previous study reported that when listening to a deceptive cadence, musicians anticipate that the music will continue, while non-musicians regard it as the ending of the music. However, there was no group difference for an authentic cadence of dominant-to-tonic [31]. Based on this, in the present study, only music-majors might be able to perceive the deceptive cadence or progressive cadence in the *Submediant*, and they might think that “the deceptive cadence thwarts the expectation for the more probable dominant-to-tonic [26]”. Therefore, we interpret that the prominent sPDC_i_ and sPDC_o_ in music-majors reflects the detection and comprehension of the more technical meaning of deceptive cadence in the *Submediant*. Thus, musical expertise in our present study does not necessarily indicate increased sensitivity or development, but a different schema based on professional training.

There is one caveat which should be mentioned. Although the ROIs in our present study include the key areas of the bilateral IFGs and STGs in music processing, it is far from depicting the whole brain network by musical expertise, experience, and harmonic cadence processing. Therefore, it warrants further study, examining the difference in effective connectivity measurements and the whole brain connectivity for musical expertise, experience, and harmonic cadence processing. Nevertheless, our data show that music-majors, with higher musical expertise, are better in identifying a less unexpected cadence than non-music-majors, with connectivity changes centered on the left IFG.

## Supporting information

S1 Table Four-way repeated measures ANOVA for the factors of Condition, Group, Site and Hemisphere. (DOCX)

S2 Table *Post hoc* for Group factor in four-way repeated measures ANOVA. (DOCX)

S3 Table *Post hoc* for Hemisphere factor in four-way repeated measures ANOVA. (DOCX)

S4 Table *Post hoc* for Condition factor in four-way repeated measures ANOVA. (DOCX)

S5 Table Three-way repeated measures ANOVAs for the factors of Condition, Group, and Flow, and *post hoc* results. (DOCX)

S6 Table Mean and SD values of Condition, Group, Site, Hemisphere, and Flow factors. (DOCX)

## References

[1] Koelsch S, Jentschke S. Short-term effects of processing musical syntax: an ERP study. Brain research. 2008;1212:55–62. Epub 2008/04/29. 10.1016/j.brainres.2007.10.078 . 18439987

[2] Kim CH, Lee S, Kim JS, Seol J, Yi SW, Chung CK. Melody effects on ERANm elicited by harmonic irregularity in musical syntax. Brain research. 2014;1560:36–45. 10.1016/j.brainres.2014.02.045 . 24607297

[3] Kim SG, Kim JS, Chung CK. The effect of conditional probability of chord progression on brain response: an MEG study. PloS one. 2011;6(2):e17337 Epub 2011/03/03. 10.1371/journal.pone.0017337 21364895 PMC3045443

[4] Koelsch S, Gunter T, Friederici AD, Schroger E. Brain indices of music processing: "nonmusicians" are musical. Journal of cognitive neuroscience. 2000;12(3):520–41. Epub 2000/08/10. 10.1162/089892900562183 . 10931776

[5] Rohrmeier MA. A generative grammar approach to diatonic harmonic structure. Proceedings SMC'07, 4th Sound and Music Computing Conference. 2007.

[6] Murphy HA, Stringham EJ. Creative harmony and musicianship; an introduction to the structure of music New York,: Prentice-Hall; 1951. xix, 618 p. p.

[7] Piston W, DeVoto M. Harmony. 5th ed New York: Norton; 1987 xvi, 575 p. p.

[8] Baccala LA, Sameshima K. Partial directed coherence: a new concept in neural structure determination. Biological cybernetics. 2001;84(6):463–74. Epub 2001/06/22. 10.1007/PL00007990 . 11417058

[9] Taulu S, Simola J. Spatiotemporal signal space separation method for rejecting nearby interference in MEG measurements. Physics in medicine and biology. 2006;51(7):1759–68. Epub 2006/03/23. 10.1088/0031-9155/51/7/008 . 16552102

[10] Taulu S, Hari R. Removal of magnetoencephalographic artifacts with temporal signal-space separation: demonstration with single-trial auditory-evoked responses. Human brain mapping. 2009;30(5):1524–34. Epub 2008/07/29. 10.1002/hbm.20627 . 18661502 PMC6871056

[11] Maess B, Koelsch S, Gunter TC, Friederici AD. Musical syntax is processed in Broca's area: an MEG study. Nature neuroscience. 2001;4(5):540–5. 10.1038/87502 . 11319564

[12] Koelsch S, Jentschke S. Differences in electric brain responses to melodies and chords. Journal of cognitive neuroscience. 2010;22(10):2251–62. Epub 2009/08/26. 10.1162/jocn.2009.21338 . 19702466

[13] Patterson RD, Uppenkamp S, Johnsrude IS, Griffiths TD. The processing of temporal pitch and melody information in auditory cortex. Neuron. 2002;36(4):767–76. Epub 2002/11/21. 10.1016/s0896-6273(02)01060-7 . 12441063

[14] Dohn A, Garza-Villarreal EA, Chakravarty MM, Hansen M, Lerch JP, Vuust P. Gray- and white-matter anatomy of absolute pitch possessors. Cereb Cortex. 2015;25(5):1379–88. 10.1093/cercor/bht334 . 24304583

[15] Schneider P, Sluming V, Roberts N, Scherg M, Goebel R, Specht HJ, et al Structural and functional asymmetry of lateral Heschl's gyrus reflects pitch perception preference. Nature neuroscience. 2005;8(9):1241–7. Epub 2005/08/24. 10.1038/nn1530 . 16116442

[16] Janata P, Birk JL, Van Horn JD, Leman M, Tillmann B, Bharucha JJ. The cortical topography of tonal structures underlying Western music. Science. 2002;298(5601):2167–70. Epub 2002/12/14. 10.1126/science.1076262 . 12481131

[17] Sammler D, Koelsch S, Ball T, Brandt A, Elger CE, Friederici AD, et al Overlap of musical and linguistic syntax processing: intracranial ERP evidence. Annals of the New York Academy of Sciences. 2009;1169:494–8. Epub 2009/08/14. 10.1111/j.1749-6632.2009.04792.x . 19673829

[18] Patel AD, Balaban E. Human pitch perception is reflected in the timing of stimulus-related cortical activity. Nature neuroscience. 2001;4(8):839–44. 10.1038/90557 . 11477431

[19] Sammler D, Koelsch S, Friederici AD. Are left fronto-temporal brain areas a prerequisite for normal music-syntactic processing? Cortex; a journal devoted to the study of the nervous system and behavior. 2011;47(6):659–73. Epub 2010/06/24. 10.1016/j.cortex.2010.04.007 . 20570253

[20] Obleser J, Meyer L, Friederici AD. Dynamic assignment of neural resources in auditory comprehension of complex sentences. NeuroImage. 2011;56(4):2310–20. 10.1016/j.neuroimage.2011.03.035 . 21421059

[21] Nan Y, Knosche TR, Zysset S, Friederici AD. Cross-cultural music phrase processing: an fMRI study. Human brain mapping. 2008;29(3):312–28. 10.1002/hbm.20390 . 17497646 PMC6871102

[22] Evers S, Dannert J, Rodding D, Rotter G, Ringelstein EB. The cerebral haemodynamics of music perception. A transcranial Doppler sonography study. Brain: a journal of neurology. 1999;122 (Pt 1):75–85. 10.1093/brain/122.1.75 . 10050896

[23] Loui P, Li HC, Hohmann A, Schlaug G. Enhanced cortical connectivity in absolute pitch musicians: a model for local hyperconnectivity. Journal of cognitive neuroscience. 2011;23(4):1015–26. Epub 2010/06/03. 10.1162/jocn.2010.21500 20515408 PMC3012137

[24] Jancke L, Langer N, Hanggi J. Diminished whole-brain but enhanced peri-sylvian connectivity in absolute pitch musicians. Journal of cognitive neuroscience. 2012;24(6):1447–61. Epub 2012/04/25. 10.1162/jocn_a_00227 . 22524277

[25] Bhattacharya J, Petsche H. Phase synchrony analysis of EEG during music perception reveals changes in functional connectivity due to musical expertise. Signal Processing. 2005;85(11):2161–77. 10.1016/j.sigpro.2005.07.007

[26] Huron DB. Sweet anticipation: music and the psychology of expectation Cambridge, Mass: MIT Press; 2006. xii, 462 p. p.

[27] James CE, Britz J, Vuilleumier P, Hauert CA, Michel CM. Early neuronal responses in right limbic structures mediate harmony incongruity processing in musical experts. NeuroImage. 2008;42(4):1597–608. Epub 2008/07/22. 10.1016/j.neuroimage.2008.06.025 . 18640279

[28] Biancorosso G. Whose Phenomenology of Music? David Huron's Theory of Expectation. Music and Letters. 2008;89(3):396–404. 10.1093/ml/gcn015

[29] Koelsch S, Schmidt BH, Kansok J. Effects of musical expertise on the early right anterior negativity: an event-related brain potential study. Psychophysiology. 2002;39(5):657–63. Epub 2002/09/19. doi: 10.1017.S0048577202010508 . 12236333 10.1017/S0048577202010508

[30] Jentschke S, Koelsch S. Musical training modulates the development of syntax processing in children. NeuroImage. 2009;47(2):735–44. Epub 2009/05/12. 10.1016/j.neuroimage.2009.04.090 . 19427908

[31] Sears D, Caplin WE, McAdams S. Perceiving the Classical Cadence. Music Perception: An Interdisciplinary Journal. 2014;31(5):397–417. 10.1525/mp.2014.31.5.397

