# Correction: Change in left inferior frontal connectivity with less unexpected harmonic cadence by musical expertise

**DOI:** 10.1371/journal.pone.0226296

**Published:** 2019-12-05

**Authors:** The PLOS ONE Staff

The following information is missing from the Funding statement: This study was also supported by the Brain Research Program through the National Research Foundation of Korea (NRF), funded by the Ministry of Science and ICT (2016M3C7A1904984). The publisher apologizes for the errors.

## References

[1] Kim CH, Kim JS, Choi Y, Kyong J-S, Kim Y, Yi SW, et al (2019) Change in left inferior frontal connectivity with less unexpected harmonic cadence by musical expertise. PLoS ONE 14(11): e0223283 10.1371/journal.pone.0223283 31714920 PMC6850538

