# Supplementary material for: Change in left inferior frontal connectivity with less unexpected harmonic cadence by musical expertise
Source: PLoS One. 2019 Nov 12;14(11):e0223283. doi: 10.1371/journal.pone.0223283 (PMC6850538; doi:10.1371/journal.pone.0223283)
Supplement: S1 Table — (DOCX) [file pone.0223283.s001.docx]

**S1 Table. Four-way repeated measures ANOVA for the factors of Condition, Group, Site and Hemisphere.** The significant *P*-values were marked in bold letters (* *p* < 0.05, ** *p* < 0.01, and *** *p* < 0.001).

|  | ***Inflow*** | | ***Outflow*** | |
| --- | --- | --- | --- | --- |
|  | ***F*** | ***P*** | ***F*** | ***P*** |
| ***Condition*** | 0.004 | 0.996 | 0.034 | 0.967 |
| ***Condition*** $\boldsymbol{\times}$ ***Group*** | 0.044 | 0.957 | 0.377 | 0.686 |
| ***Condition*** $\boldsymbol{\times}$ ***Site*** | 3.059 | 0.050 | 6.054 | **0.003 **** |
| ***Condition*** $\boldsymbol{\times}$ ***Hemisphere*** | 5.037 | **0.008 **** | 3.364 | **0.037 *** |
| ***Condition*** $\boldsymbol{\times}$ ***Group*** $\boldsymbol{\times}$ ***Site*** | 3.584 | **0.030 *** | 4.295 | **0.016 *** |
| ***Condition*** $\boldsymbol{\times}$ ***Group*** $\boldsymbol{\times}$ ***Hemisphere*** | 6.052 | **0.003**** | 3.474 | **0.034 *** |
| ***Condition*** $\boldsymbol{\times}$ ***Site*** $\boldsymbol{\times}$ ***Hemisphere*** | 5.722 | **0.004 **** | 4.832 | **0.009 **** |
| ***Condition*** $\boldsymbol{\times}$ ***Group*** $\boldsymbol{\times}$ ***Site*** $\boldsymbol{\times}$ ***Hemisphere*** | 5.963 | **0.003 **** | 5.681 | **0.004 **** |
| ***Group*** | 0.000 | 0.993 | 0.001 | 0.981 |
| ***Site*** | 22.734 | 0.993 | 25.557 | **0.000003***** |
| ***Hemisphere*** | 5.283 | **0.0001 ***** | 2.767 | 0.101 |
| ***Group*** $\boldsymbol{\times}$ ***Site*** | 0.062 | **0.025 *** | 0.274 | 0.602 |
| ***Group*** $\boldsymbol{\times}$ ***Hemispher*** | 5.422 | 0.805 | 6.783 | **0.011*** |
| ***Site*** $\boldsymbol{\times}$ ***Hemisphere*** | 3.664 | **0.023 *** | 3.425 | 0.069 |
| ***Group*** $\boldsymbol{\times}$ ***Site*** $\boldsymbol{\times}$ ***Hemisphere*** | 7.002 | 0.060 | 8.982 | **0.004 **** |
