# Supplementary material for: Change in left inferior frontal connectivity with less unexpected harmonic cadence by musical expertise
Source: PLoS One. 2019 Nov 12;14(11):e0223283. doi: 10.1371/journal.pone.0223283 (PMC6850538; doi:10.1371/journal.pone.0223283)
Supplement: S2 Table — (DOCX) [file pone.0223283.s002.docx]

**S2 Table. *Post hoc* for Group factor in four-way repeated measures ANOVA.** The significant *P*-values were marked in bold letters. In *post hoc* *t* test for 3 conditions $\times$ 2 sites $\times$ 2 hemispheres, the significance levels of *P*-values adjusted by the Bonferroni test are * *p* < 0.05, ** *p* < 0.01, and *** *p* < 0.001. The uncorrected *P*-values are also presented.

|  |  | ***Inflow*** | | | ***Outflow*** | | |
| --- | --- | --- | --- | --- | --- | --- | --- |
|  |  | ***t*** | ***P (uncorrected)*** | ***P (corrected)*** | ***t*** | ***P (uncorrected)*** | ***P (corrected)*** |
| ***Left IFG*** | ***Tonic*** | -1.208 | 0.244 | 1.0 | 0.864 | 0.4 | 1.0 |
|  | ***Submediant*** | 4.934 | **0.0001 **** | **0.002 **** | -4.391 | **0.0004 **** | **0.005 **** |
|  | ***Supertonic*** | 1.809 | 0.088 | 1.0 | -1.145 | 0.268 | 1.0 |
| ***Right IFG*** | ***Tonic*** | 0.244 | 0.810 | 1.0 | 0.240 | 0.813 | 1.0 |
|  | ***Submediant*** | -2.456 | 0.025 | 0.301 | 2.531 | 0.022 | 0.258 |
|  | ***Supertonic*** | -1.893 | 0.075 | 0.905 | 2.581 | 0.019 | 0.233 |
| ***Left STG*** | ***Tonic*** | 0.500 | 0.623 | 1.0 | -1.565 | 0.136 | 1.0 |
|  | ***Submediant*** | -1.941 | 0.069 | 0.828 | 2.447 | 0.026 | 0.307 |
|  | ***Supertonic*** | 0.247 | 0.808 | 1.0 | -0.206 | 0.839 | 1.0 |
| ***Right STG*** | ***Tonic*** | 1.022 | 0.321 | 1.0 | -1.386 | 0.184 | 1.0 |
|  | ***Submediant*** | -0.991 | 0.335 | 1.0 | 0.758 | 0.459 | 1.0 |
|  | ***Supertonic*** | 0.339 | 0.739 | 1.0 | -0.494 | 0.628 | 1.0 |
