# Supplementary material for: Change in left inferior frontal connectivity with less unexpected harmonic cadence by musical expertise
Source: PLoS One. 2019 Nov 12;14(11):e0223283. doi: 10.1371/journal.pone.0223283 (PMC6850538; doi:10.1371/journal.pone.0223283)
Supplement: S3 Table — (DOCX) [file pone.0223283.s003.docx]

**S3 Table. *Post hoc* for Hemisphere factor in four-way repeated measures ANOVA.** The significant *P*-values were marked in bold letters. In *post hoc* *t* test for 3 conditions $\times$ 2 sites $\times$ 2 hemispheres, the significance levels of *P*-values adjusted by the Bonferroni test are * *p* < 0.05 and ** *p* < 0.01. Also, the uncorrected *P*-values are presented.

|  |  |  | ***Inflow*** | | | ***Outflow*** | | |
| --- | --- | --- | --- | --- | --- | --- | --- | --- |
|  |  |  | ***t*** | ***P (uncorrected)*** | ***P (corrected)*** | ***t*** | ***P (uncorrected)*** | ***P (corrected)*** |
| ***Music-majors*** | ***IFG*** | ***Tonic*** | 0.834 | 0.429 | 1.0 | -0.774 | 0.461 | 1.0 |
|  |  | ***Submediant*** | 5.262 | **0.00076 **** | **0.009 **** | -4.889 | **0.001 *** | **0.015 *** |
|  |  | ***Supertonic*** | 0.837 | 0.427 | 1.0 | -1.021 | 0.337 | 1.0 |
|  | ***STG*** | ***Tonic*** | 1.425 | 0.192 | 1.0 | -1.247 | 0.248 | 1.0 |
|  |  | ***Submediant*** | -1.217 | 0.258 | 1.0 | 1.332 | 0.219 | 1.0 |
|  |  | ***Supertonic*** | 0.043 | 0.967 | 1.0 | 0.249 | 0.809 | 1.0 |
| ***Non-music-majors*** | ***IFG*** | ***Tonic*** | 2.022 | 0.074 | 0.886 | -1.423 | 0.188 | 1.0 |
|  |  | ***Submediant*** | -0.674 | 0.517 | 1.0 | 0.691 | 0.507 | 1.0 |
|  |  | ***Supertonic*** | -2.410 | 0.039 | 0.471 | 2.151 | 0.060 | 0.719 |
|  | ***STG*** | ***Tonic*** | 1.585 | 0.147 | 1.0 | -0.868 | 0.408 | 1.0 |
|  |  | ***Submediant*** | -0.622 | 0.549 | 1.0 | 0.380 | 0.713 | 1.0 |
|  |  | ***Supertonic*** | 0.096 | 0.926 | 1.0 | -0.013 | 0.990 | 1.0 |
