# Supplementary material for: Change in left inferior frontal connectivity with less unexpected harmonic cadence by musical expertise
Source: PLoS One. 2019 Nov 12;14(11):e0223283. doi: 10.1371/journal.pone.0223283 (PMC6850538; doi:10.1371/journal.pone.0223283)
Supplement: S4 Table — (DOCX) [file pone.0223283.s004.docx]

**S4 Table. *Post hoc* for Condition factor in four-way repeated measures ANOVA.** The significant *P*-values were marked in bold letters. In *post hoc* one-way ANOVAs for 2 groups $\times$ 2 hemispheres $\times$ 2 sites and in paired *t* test for 3 conditions, the significance levels of *P*-values corrected by the Bonferroni test are * *p* < 0.05 and ** *p* < 0.01. The uncorrected *P*-values are also presented.

|  | |  | | ***Inflow*** | | | | ***Outflow*** | | |
| --- | --- | --- | --- | --- | --- | --- | --- | --- | --- | --- |
|  | | |  | | ***F/t*** | ***P (uncorrected)*** | ***P (corrected)*** | ***F/t*** | ***P (uncorrected)*** | ***P (corrected)*** |
| ***Music-majors*** | ***Left IFG*** | | ***Condition effect*** | | 7.583 | **0.005 *** | **0.039 *** | 8.969 | **0.002 **** | **0.020 **** |
|  |  | | ***Tonic vs. Submediant*** | | -3.153 | **0.014 *** | **0.041 *** | 2.921 | 0.019 | 0.058 |
|  |  | | ***Submediant vs. Supertonic*** | | 4.509 | **0.002 **** | **0.006 **** | -4.143 | **0.003 *** | **0.010 *** |
|  |  | | ***Tonic vs. Supertonic*** | | 0.350 | 0.736 | 1.0 | -1.151 | 0.283 | 0.849 |
|  | ***Right IFG*** | | ***Condition effect*** | | 1.106 | 0.330 | 1.0 | 1.836 | 0.210 | 0.840 |
|  | ***Left STG*** | | ***Condition effect*** | | 3.559 | 0.053 | 0.211 | 4.563 | 0.027 | 0.108 |
|  | ***Right STG*** | | ***Condition effect*** | | 1.340 | 0.290 | 1.158 | 0.941 | 0.411 | 1.0 |
|  |  | |  | |  |  |  |  |  |  |
| ***Non-music-majors*** | ***Left IFG*** | | ***Condition effect*** | | 9.825 | **0.001 **** | **0.010 **** | 7.987 | **0.003 *** | **0.026 *** |
|  |  | | ***Tonic vs. Submediant*** | | 2.761 | 0.022 | 0.066 | -2.618 | 0.028 | 0.084 |
|  |  | | ***Submediant vs. Supertonic*** | | 1.494 | 0.169 | 0.508 | -1.311 | 0.222 | 0.667 |
|  |  | | ***Tonic vs. Supertonic*** | | 4.151 | **0.002 **** | **0.007 **** | -3.887 | **0.004 **** | **0.011 *** |
|  | ***Right IFG*** | | ***Condition effect*** | | 1.923 | 0.175 | 0.700 | 0.704 | 0.508 | 1.0 |
|  | ***Left STG*** | | ***Condition effect*** | | 0.125 | 0.883 | 1.0 | 1.199 | 0.325 | 1.0 |
|  | ***Right STG*** | | ***Condition effect*** | | 2.493 | 0.137 | 0.547 | 2.681 | 0.096 | 0.383 |
