# Supplementary material for: Change in left inferior frontal connectivity with less unexpected harmonic cadence by musical expertise
Source: PLoS One. 2019 Nov 12;14(11):e0223283. doi: 10.1371/journal.pone.0223283 (PMC6850538; doi:10.1371/journal.pone.0223283)
Supplement: S5 Table — (DOCX) [file pone.0223283.s005.docx]

**S5 Table. Three-way repeated measures ANOVAs for the factors of Condition, Group, and Flow, and *post hoc* results.** The significant *P*-values were marked in bold letters. In thee-way ANOVAs for 2 groups $\times$ 2 flows, and in *post hoc* paired *t* teat for 2 groups $\times$ 3 conditions, the significance levels of *P*-values corrected by the Bonferroni test are * *p* < 0.05, ** *p* < 0.001, and *** *p* < 0.001.

|  |  | | ***F/t*** | ***P (uncorrected)*** | ***P (corrected)*** |
| --- | --- | --- | --- | --- | --- |
| ***Three-way ANOVA***  ***in the left IFG*** | ***Condition*** | | 2.859 | 0.064 | 0.257 |
|  | ***Condition*** $\boldsymbol{\times}$ ***Group*** | | 4.634 | 0.013 | 0.052 |
|  | ***Condition*** $\boldsymbol{\times}$ ***Flow*** | | 12.442 | **0.00002 ***** | **0.0001 ***** |
|  | ***Condition*** $\boldsymbol{\times}$ ***Group*** $\boldsymbol{\times}$ ***Flow*** | | 14.661 | **0.000005 ***** | **0.00002 ***** |
|  | ***Group*** | | 1.632 | 0.210 | 0.840 |
|  | ***Flow*** | | 18.875 | **0.0001 **** | **0.0005 **** |
|  | ***Group*** $\boldsymbol{\times}$ ***Flow*** | | 6.443 | 0.016 | 0.636 |
|  |  | |  |  |  |
| ***Three-way ANOVA***  ***in the right IFG*** | ***Condition*** | | 0.758 | 0.445 | 1.0 |
|  | ***Condition*** $\boldsymbol{\times}$ ***Group*** | | 0.730 | 0.456 | 1.0 |
|  | ***Condition*** $\boldsymbol{\times}$ ***Flow*** | | 1.588 | 0.216 | 0.865 |
|  | ***Condition*** $\boldsymbol{\times}$ ***Group*** $\boldsymbol{\times}$ ***Flow*** | | 2.880 | 0.077 | 0.306 |
|  | ***Group*** | | 1.703 | 0.201 | 0.803 |
|  | ***Flow*** | | 0.292 | 0.593 | 1.0 |
|  | ***Group*** $\boldsymbol{\times}$ ***Flow*** | | 11.583 | **0.002 **** | **0.007 **** |
|  |  | |  |  |  |
| ***Three-way ANOVA***  ***in the left STG*** | ***Condition*** | | 0.812 | 0.448 | 0.448 |
|  | ***Condition*** $\boldsymbol{\times}$ ***Group*** | | 0.276 | 0.760 | 0.760 |
|  | ***Condition*** $\boldsymbol{\times}$ ***Flow*** | | 3.094 | 0.052 | 0.207 |
|  | ***Condition*** $\boldsymbol{\times}$ ***Group*** $\boldsymbol{\times}$ ***Flow*** | | 2.539 | 0.086 | 0.346 |
|  | ***Group*** | | 0.113 | 0.739 | 1.0 |
|  | ***Flow*** | | 12.196 | **0.001 ***** | **0.005 ***** |
|  | ***Group*** $\boldsymbol{\times}$ ***Flow*** | | 0.087 | 0.770 | 1.0 |
|  |  | |  |  |  |
| ***Three-way ANOVA***  ***in the right STG*** | ***Condition*** | | 0.986 | 0.360 | 1.0 |
|  | ***Condition*** $\boldsymbol{\times}$ ***Group*** | | 0.322 | 0.666 | 1.0 |
|  | ***Condition*** $\boldsymbol{\times}$ ***Flow*** | | 4.601 | 0.022 | 0.090 |
|  | ***Condition*** $\boldsymbol{\times}$ ***Group*** $\boldsymbol{\times}$ ***Flow*** | | 1.800 | 0.183 | 0.730 |
|  | ***Group*** | | 0.016 | 0.900 | 1.0 |
|  | ***Flow*** | | 14.477 | **0.0006 **** | **0.002 **** |
|  | ***Group*** $\boldsymbol{\times}$ ***Flow*** | | 0.046 | 0.832 | 1.0 |
|  |  | |  |  |  |
| ***Post hoc***  ***paired t test***  ***(Flow difference***  ***in the left IFG)*** | ***Music-majors*** | ***Tonic*** | 1.631 | 0.142 | 0.849 |
|  |  | ***Submediant*** | 5.929 | **0.00035 **** | **0.002 **** |
|  |  | ***Supertonic*** | 0.829 | 0.431 | 0.257 |
|  | ***Non-Music-majors*** | ***Tonic*** | 2.994 | 0.015 | 0.091 |
|  |  | ***Submediant*** | 0.086 | 0.934 | 1.0 |
|  |  | ***Supertonic*** | -1.561 | 0.153 | 0.917 |
|  |  |  |  |  |  |
| ***Post hoc***  ***paired t test***  ***(Flow difference***  ***in the right IFG)*** | ***Music-majors*** | ***Tonic*** | 0.175 | 0.865 | 1.0 |
|  |  | ***Submediant*** | -3.784 | **0.005 *** | **0.032 *** |
|  |  | ***Supertonic*** | -0.657 | 0.530 | 1.0 |
|  | ***Non-Music-majors*** | ***Tonic*** | 0.030 | 0.977 | 0.091 |
|  |  | ***Submediant*** | 0.989 | 0.349 | 1.0 |
|  |  | ***Supertonic*** | 2.226 | 0.053 | 0.917 |
