# Supplementary material for: Change in left inferior frontal connectivity with less unexpected harmonic cadence by musical expertise
Source: PLoS One. 2019 Nov 12;14(11):e0223283. doi: 10.1371/journal.pone.0223283 (PMC6850538; doi:10.1371/journal.pone.0223283)
Supplement: S6 Table — (DOCX) [file pone.0223283.s006.docx]

**S6 Table. Mean and SD values of Condition, Group, Site, Hemisphere, and Flow factors.**

|  | ***N*** | ***Mean*** | ***SD*** |
| --- | --- | --- | --- |
| ***T_lSTG_IN_M*** | 9 | 1.400036 | 0.477010 |
| ***SM_lSTG_IN_M*** | 9 | 0.706820 | 0.417817 |
| ***ST_lSTG_IN_M*** | 9 | 1.521420 | 1.088662 |
| ***T_rSTG_IN_M*** | 9 | 1.002499 | 0.799898 |
| ***SM_rSTG_IN_M*** | 9 | 1.097647 | 0.603461 |
| ***ST_rSTG_IN_M*** | 9 | 1.499172 | 0.894929 |
| ***T_lIFG_IN_M*** | 9 | 2.146640 | 1.268493 |
| ***SM_lIFG_IN_M*** | 9 | 3.681923 | 1.110632 |
| ***ST_lIFG_IN_M*** | 9 | 1.946339 | 1.220911 |
| ***T_rIFG_IN_M*** | 9 | 1.585860 | 1.129550 |
| ***SM_rIFG_IN_M*** | 9 | 0.837060 | 0.532526 |
| ***ST_rIFG_IN_M*** | 9 | 1.373798 | 1.172438 |
| ***T_lSTG_OUT_M*** | 9 | 1.595802 | 0.281223 |
| ***SM_lSTG_OUT_M*** | 9 | 1.891952 | 0.123766 |
| ***ST_lSTG_OUT_M*** | 9 | 1.590473 | 0.344662 |
| ***T_rSTG_OUT_M*** | 9 | 1.718774 | 0.336342 |
| ***SM_rSTG_OUT_M*** | 9 | 1.726850 | 0.339374 |
| ***ST_rSTG_OUT_M*** | 9 | 1.546743 | 0.326427 |
| ***T_lIFG_OUT_M*** | 9 | 1.324427 | 0.301756 |
| ***SM_lIFG_OUT_M*** | 9 | 0.860646 | 0.354648 |
| ***ST_lIFG_OUT_M*** | 9 | 1.504351 | 0.398369 |
| ***T_rIFG_OUT_M*** | 9 | 1.496030 | 0.425360 |
| ***SM_rIFG_OUT_M*** | 9 | 1.843997 | 0.294756 |
| ***ST_rIFG_OUT_M*** | 9 | 1.699161 | 0.326967 |
| ***T_lSTG_IN_NM*** | 10 | 1.232444 | 0.895089 |
| ***SM_lSTG_IN_NM*** | 10 | 1.237967 | 0.717287 |
| ***ST_lSTG_IN_NM*** | 10 | 1.399219 | 1.066591 |
| ***T_rSTG_IN_NM*** | 10 | 0.715746 | 0.368674 |
| ***SM_rSTG_IN_NM*** | 10 | 1.496254 | 1.059871 |
| ***ST_rSTG_IN_NM*** | 10 | 1.353678 | 0.966348 |
| ***T_lIFG_IN_NM*** | 10 | 2.920810 | 1.498820 |
| ***SM_lIFG_IN_NM*** | 10 | 1.568667 | 0.738292 |
| ***ST_lIFG_IN_NM*** | 10 | 1.020421 | 1.009423 |
| ***T_rIFG_IN_NM*** | 10 | 1.468270 | 0.972234 |
| ***SM_rIFG_IN_NM*** | 10 | 1.905922 | 1.201082 |
| ***ST_rIFG_IN_NM*** | 10 | 2.465637 | 1.324099 |
| ***T_lSTG_OUT_NM*** | 10 | 1.809086 | 0.309491 |
| ***SM_lSTG_OUT_NM*** | 10 | 1.662757 | 0.254740 |
| ***ST_lSTG_OUT_NM*** | 10 | 1.622297 | 0.327371 |
| ***T_rSTG_OUT_NM*** | 10 | 1.891599 | 0.196394 |
| ***SM_rSTG_OUT_NM*** | 10 | 1.608568 | 0.339853 |
| ***ST_rSTG_OUT_NM*** | 10 | 1.624328 | 0.354931 |
| ***T_lIFG_OUT_NM*** | 10 | 1.179898 | 0.411952 |
| ***SM_lIFG_OUT_NM*** | 10 | 1.540231 | 0.320232 |
| ***ST_lIFG_OUT_NM*** | 10 | 1.717600 | 0.411590 |
| ***T_rIFG_OUT_NM*** | 10 | 1.456684 | 0.282845 |
| ***SM_rIFG_OUT_NM*** | 10 | 1.397257 | 0.448888 |
| ***ST_rIFG_OUT_NM*** | 10 | 1.274729 | 0.383299 |

*Abbreviations*: T = Tonic, SM = Submediant, ST = Supertonic, lSTG = left STG, rSTG = right STG, lIFG = left IFG, rIFG = right IFG, IN = Inflow, OUT = Outflow, M = Music-majors, NM = Non-music-majors.
